# Bio-inspired adhesion control with liquids

**DOI:** 10.1016/j.isci.2022.103864

**Published:** 2022-02-04

**Authors:** Yupeng Chen, Zhongpeng Zhu, Martin Steinhart, Stanislav N. Gorb

**Affiliations:** 1 Key Laboratory of Bio-inspired Smart Interfacial Science and Technology of Ministry of Education, School of Chemistry, Beihang University, Beijing 100191, P.R. China; 2 Institut für Chemie neuer Materialien and CellNanOs, Universität Osnabrück, Barbarastr. 7, 49069 Osnabrück, Germany; 3 Functional Morphology and Biomechanics, Zoological Institute, Kiel University, Am Botanischen Garten 9, 24118 Kiel, Germany

**Keywords:** Environmental science, Biophysics

## Abstract

Bio-inspired surfaces enabling wet adhesion management are of significant interest for applications in the field of biomedicine, as components of bionic robots and as wearable devices. In the course of biological evolution, many organisms have evolved wet adhesive surfaces with strong attachment ability. Insects enhance their adhesion on contact substrates using secreted adhesive liquids. Here we discuss concepts of bio-inspired wet adhesion. First, remaining challenges associated with the understanding and the design of biological and artificial wet adhesive systems as well as strategies to supply adhesive liquids to their contact surfaces are reviewed. Then, future directions to construct wet adhesive surfaces with liquids are discussed in detail. Finally, a model of wet adhesion management with liquids is suggested, which might help the design of next-generation bio-inspired wet adhesive surfaces.

## Graphical abstract

**Figure:**
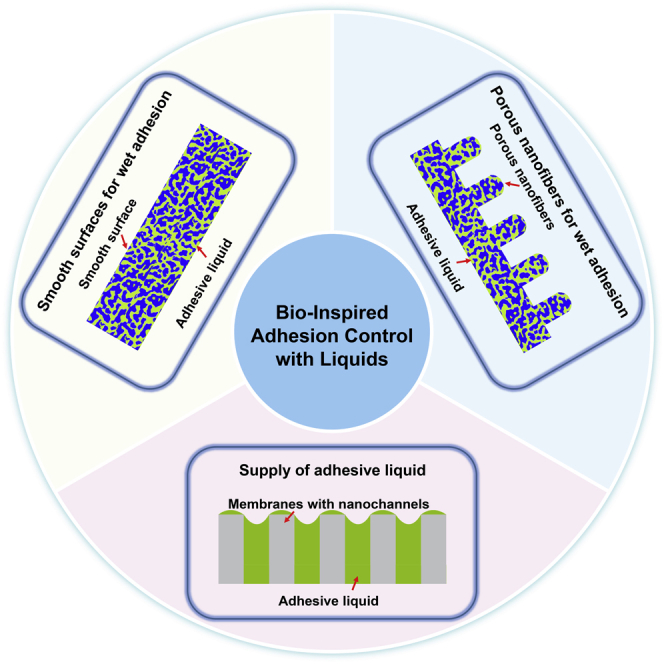

## Introduction

**Figure 1:**
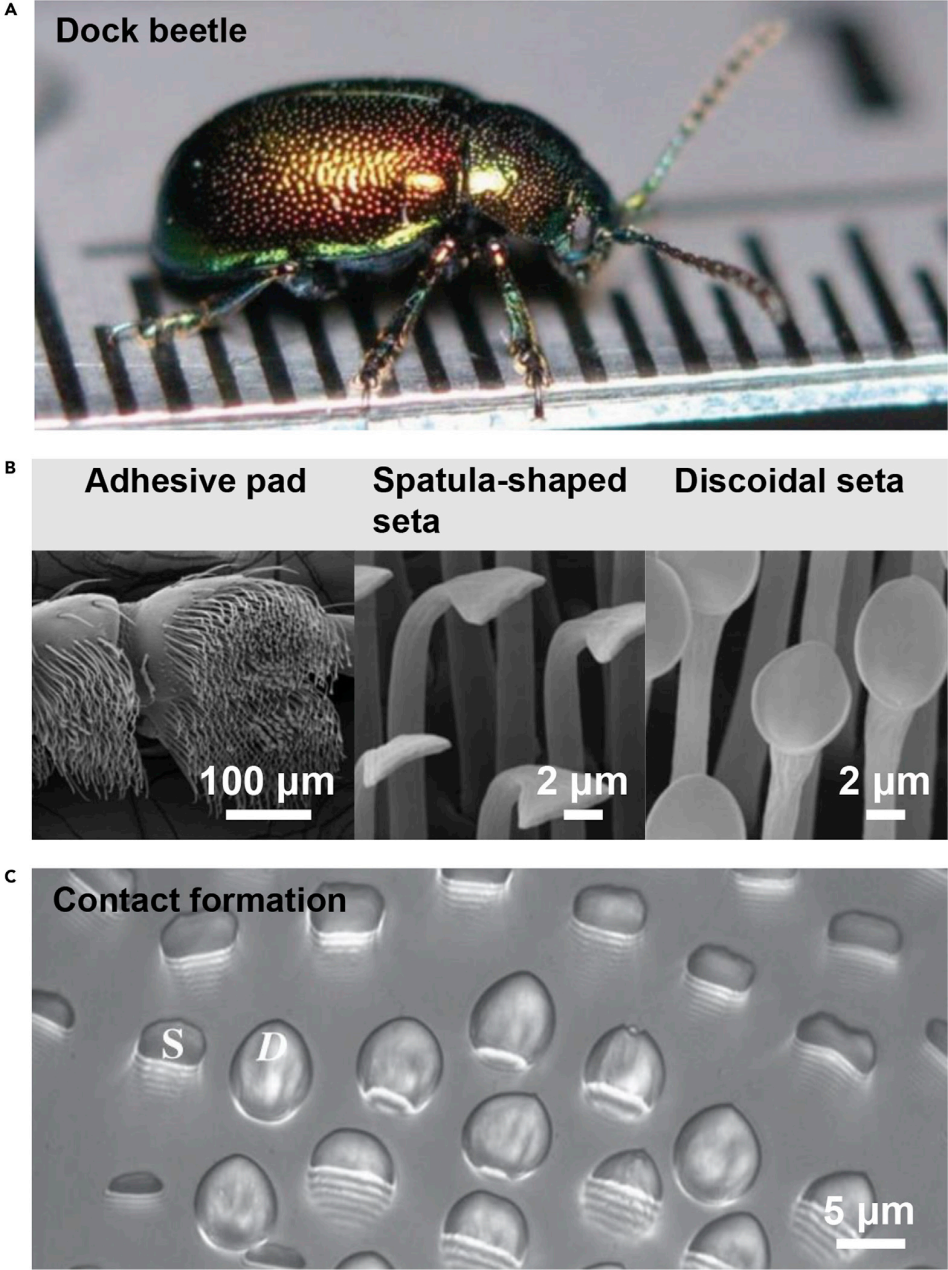
Wet adhesion control with liquids in insects (A) Macroscopic view of a dock beetle. (B) Adhesive pad covered with adhesive elements (e.g., spatula-shaped and discoidal setae). (C) Contact formation of the adhesive elements including spatula-shaped (denoted as *S*) and discoidal (denoted as *D*) setae on a glass coverslip, during which liquid bridges composed of adhesive secretions are also formed.

**Figure 2:**
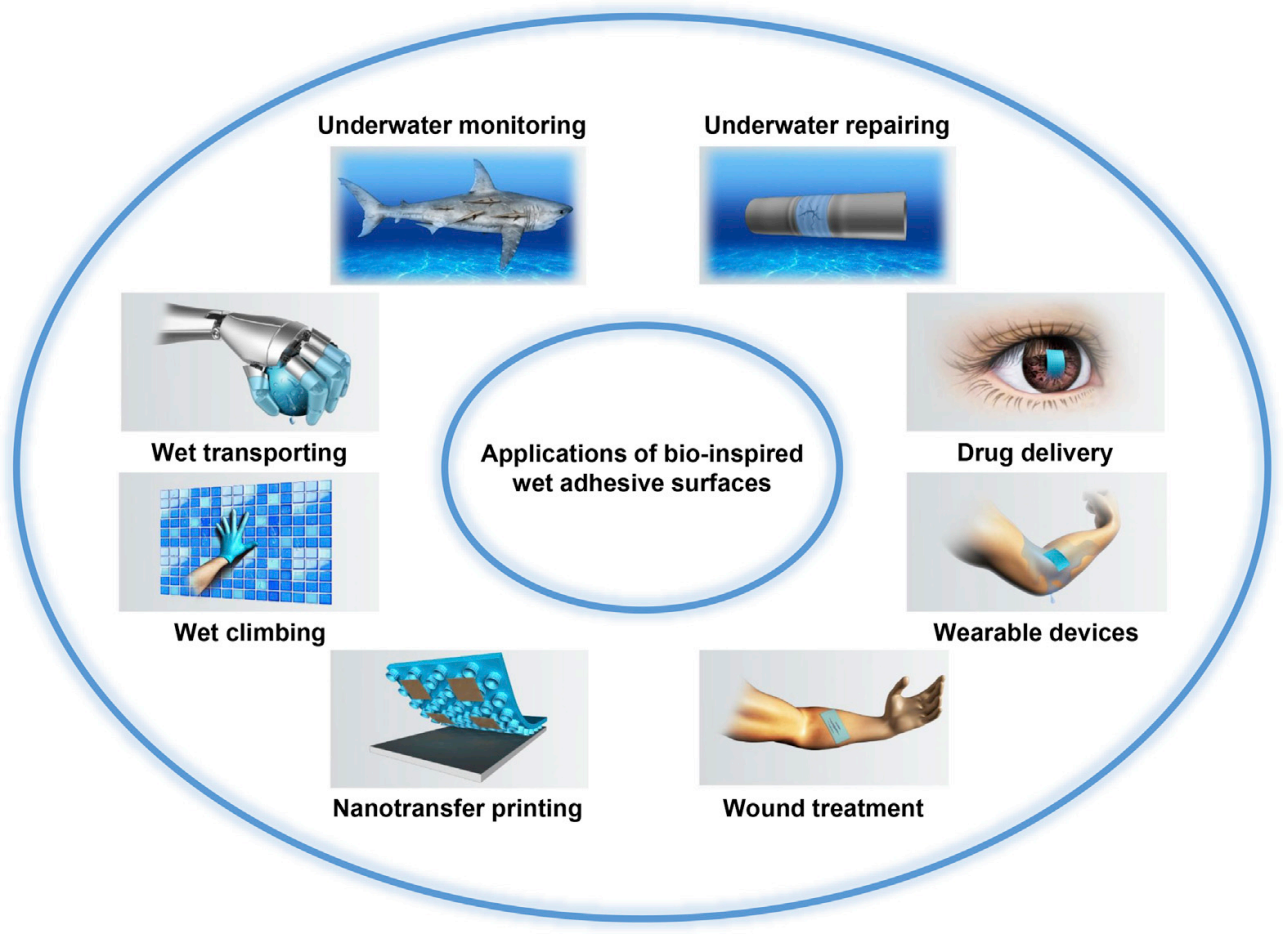
Applications of bio-inspired wet adhesive surfaces Wet adhesive surfaces can be employed for underwater repairing, portable drug delivery, wearable devices, wound care, nanotransfer printing, climbing on wet surfaces, wet transporting and underwater monitoring.

Reproduced with permission (Chen et al., 2019). Copyright 2019, Wiley-VCH

The term wet adhesion is used for a broad range of scenarios, in which adhesive organs or systems contact a counterpart surface in the presence of liquids. Adhesion under humid conditions and underwater is also considered wet adhesion. For example, insects frequently use adhesive secretions to manage adhesion on substrate surfaces. Meanwhile, it has become rather clear that liquids play a crucial role in adhesion control (Figure 1) (Dirks 2014; Dirks and Federle 2011a). During their evolution for hundreds of millions of years, many organisms have evolved unique forms of wet adhesive surfaces. Thus, it is rewarding to explore the underlying adhesion principles, which may inspire the design of bionic surfaces with similar functionalities. Bio-inspired surfaces having specific wet-adhesive properties are already used in a variety of application fields (Ahn 2017; Baik et al., 2019; Chen et al. 2018a, 2018b, 2019; Liu et al., 2021; Wang et al. 2018a, 2020b; Wang and Hensel 2021). For example, they can be employed as portable drug delivery patches for eye treatment (Than et al., 2018; Yang et al., 2013) and micromotors for stomach care (Cai et al., 2022), wearable flexible devices for the monitoring of physiological conditions of humans (Chun et al., 2018; Kim et al. 2019, 2021; Lee et al., 2021; Min et al., 2021), and as medical tapes for wound treatment (Chen and Yang 2017; Peng et al., 2021) and skin care (Baik et al., 2021). They can be also used as the adhesive pads for bionic robots, as adhesive devices for the transport of liquid droplets in intelligent manufacturing (Chen et al., 2018b; Li et al., 2020), as wet adhesives for the repair of pipes and as smart tapes for micro/nanofilm transfer (Figure 2) (Lee et al., 2016; Park et al., 2021; Yi et al., 2018). Recently, taking advantage of capillarity, patterned membranes constructed on superhydrophobic substrates were transferred to the target substrates with easy-peeling and nondestructive transfer properties, showing great potential in developing free-standing patterned membranes on large scale (Chen et al. 2021a, 2021b; Gao et al., 2021; Su and Chen 2021). Notably, surfaces with ultra-low adhesion to matter with different states were also obtained for the corresponding applications in engineering fields such as anti-fogging coatings (Guo et al., 2018; Lin et al., 2021), anti-liquid pollution (Nicolas et al., 2006), antimicrobial surfaces (Li et al., 2021; Rauner et al., 2018), anti-marine fouling (Brzozowska et al., 2014; Carve et al., 2019; Wang et al., 2020a) and anti-icing adhesion (Dhyani et al., 2021; Golovin et al., 2019; He et al., 2020; Zhang et al., 2021).

(A–C) Reproduced with permission (Gernay et al., 2016). Copyright 2016, The Author(s) Published by the Royal Society

Despite the fact that various technological applications of wet adhesion have been reported, predictive understanding of the actual function of liquids involved in wet adhesion is insufficient. Predictive understanding of wet adhesion is nevertheless the prerequisite for gaining understanding of specific adhesion mechanisms used by insects for the design of bio-inspired wet adhesive surfaces. One reason for the lack of knowledge regarding wet adhesion is that natural adhesive secretions are complex mixtures and often difficult to analyze because they are produced only in small amounts (Federle et al., 2002; Gerhardt et al., 2016; Peisker and Gorb 2012). Therefore, it is still challenging to understand how liquids at the contact interface influence adhesion, and to exploit this knowledge for the design of artificial wet adhesive surfaces. Taking advantage of the liquids with similar components and properties as adhesive secretions, artificial adhesive surfaces with specific functionalities can be obtained. For example, for some applications, it could be desirable to design artificial adhesives that show both strong normal adhesion and tangential adhesion on the surface like the wet adhesion of mussel adhesive proteins. For other applications, it might be advantageous, if moderate normal adhesion is combined with the strong shear resistance like the wet adhesion of tree frog pads. This is because different organisms adapt to their complex habitats, adult mussels usually adhere to rocks firmly for resisting the impact of waves, whereas tree frogs commonly need flexible switching between attachment and detachment for their movements.

So far, only limited research efforts have been devoted to artificial liquids for wet adhesion, which leaves large room for further exploration in the future. First, we discussed the advances of liquid-related wet adhesion. For instance, the adhesion and friction performance of the adhesive organs of cockroaches were explored in the presence of adhesive secretions ejected by the cockroaches, after the depletion of these biological secretions and in the presence of artificial secretions (i.e., squalane-based emulsions) (Betz et al., 2017). As a result, tarsal adhesion decreased about 25% after the depletion of the tarsal secretions as compared to the non-treated tarsi. In contrast, 2- to 10-fold higher adhesion was obtained after the supply of squalane and squalane-based emulsions. It is suggested that the secretions mainly work through minimizing viscous dissipation to achieve the goal of easy detachment during locomotion. On the other hand, the secretions function as a lubricant to avoid immoderate friction on both smooth and nanorough surfaces. On rougher surfaces, they can increase contact surface through maintaining the cuticle compliable and compensating surface asperities. In detail, the secretions can increase the effective contact area between the adhesive pad and the substrate through filling out surface irregularities (Drechsler and Federle 2006; Persson 2007). Furthermore, the secretions in the gaps of the soft pad cuticle can also do a favor for the viscoelastic performance (Goodwyn et al., 2006). Most recently, multiple characterization techniques were employed to investigate both surface and bulk-specific properties of artificial secretions (i.e., emulsion composed of squalane, deuterated stearic acid, and D_2_O) (Fowler et al., 2021b). First, sum-frequency generation (SFG) spectra were used to show that squalane chains exhibit similar hydrocarbon organization at the interface as natural adhesive secretions of beetles. Such surface-active hydrocarbon components in artificial and natural secretions can contribute to moderating traction and lubrication according to the contact surfaces. Rheological testing revealed that the bulk emulsion exhibited a shear-thinning profile which can enhance traction forces during locomotion. The low surface tension also ensures the emulsion can wet various hydrophobic and hydrophilic surfaces, which is required in the natural adhesion system. However, squalane-based emulsions cannot mimic the major property (i.e., low adhesion with moderate friction) of the natural adhesive secretions and their stability need to be enhanced. It is desirable to develop artificial adhesive liquids with similar components and properties to natural adhesive secretions and investigate the corresponding wet adhesion performance after the introduction of these liquids.

Considering the lack of research in the field of liquid-related wet adhesion and the promising prospects in constructing artificial adhesive surfaces, we discuss concepts of bio-inspired wet adhesion control with liquids through combining surface wetting and adhesion in this perspective (Liu et al., 2017; Su et al., 2016; Wang et al., 2019; Zhu et al. 2017, 2021). Three main aspects are reviewed—biological wet adhesive surfaces, artificial wet adhesive surfaces, and the supply of adhesive liquids to the contact surfaces of adhesive systems. After that, potential future directions are presented. It is anticipated that the regulation of wetting and adhesion on contact surfaces can be investigated through the selection of model liquids, which further provides guidance for the design of artificial wet adhesive surfaces with specific functionalities.

### Main challenges

#### Biological wet adhesive surfaces

**Figure 3:**
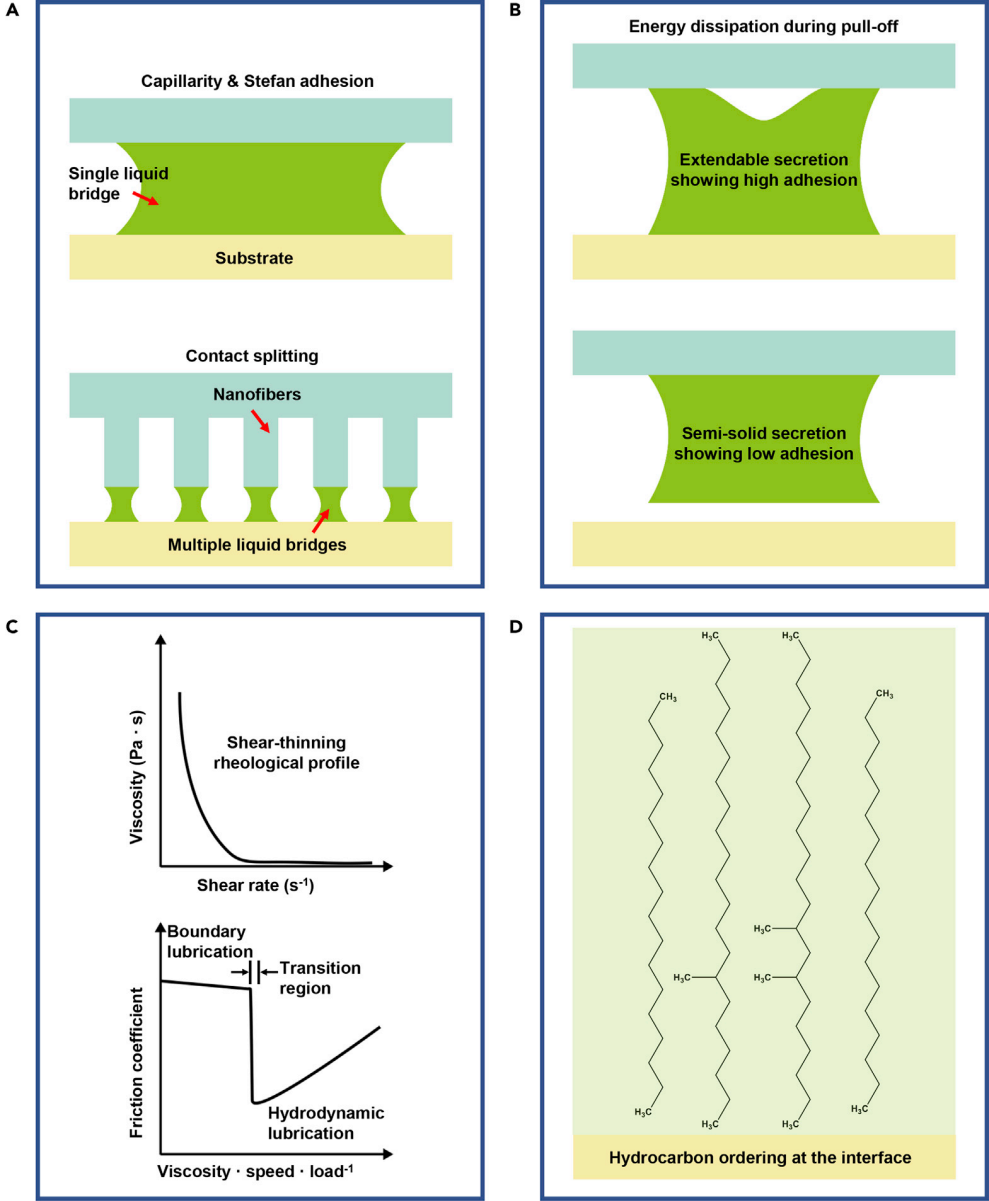
Main principles of wet adhesion (A) Capillarity and Stefan adhesion working through the single liquid bridge between the adhesive elements and the contact substrates (top) and contact splitting model after considering the multiple liquid bridges (bottom). (B) Energy dissipation systems during pull-off when extendable secretions (top) and semi-solid secretions (bottom) are considered, respectively. (C) Shear-thinning property of the secretions (top) and boundary lubrication (bottom) contribute to enhancing friction. (D) Dynamic molecular ordering at the contact surface contributes to mediating wet adhesion. The adhesive element, contact surface and adhesive liquid are represented by the cyan, yellow and green colors, respectively

First, the main principles of wet adhesion were introduced concisely according to previous research. Capillarity is related to the presence of curved liquid surfaces in the vicinity of a solid surface. The main parameters influencing capillarity are the surface tension of the considered fluid and the curvatures of the fluid interfaces (e.g., the geometry of liquid bridges is characterized by two principal curvatures) (Butt et al., 2010; Zakerin et al., 2013). Besides the Laplace pressure related to the presence of curved liquid interfaces, viscous interactions (e.g., Stefan adhesion) contribute to perpendicular adhesion (top in Figure 3A) (Dirks 2014). Considering the hierarchical structures of adhesive organs for decreasing the size of adhesive elements in nature, an extended contact splitting model was proposed (bottom in Figure 3A) (De Souza et al., 2008; Labonte and Federle 2015). When the contact angle (*θ*) on the substrates is less than 30°, contact splitting can decrease adhesion, when 90° < *θ <* 150°, contact splitting can increase adhesion. When 30° < *θ* < 90°, the generated adhesion will depend on the specific variables (e.g., number of adhesive elements, area coverage, and contact angle). For normal adhesion, two different energy dissipation systems were given (Betz et al., 2017). Adhesive strength can be increased taking advantage of viscous and viscoelastic dissipation from extendable secretions and pad cuticles, respectively (top in Figure 3B). Whereas low adhesion can be obtained when the stiff pad cuticle was covered by semi-solid secretions (bottom in Figure 3B). The surface tension can contribute to the tangential adhesion (i.e., friction) partly and be enhanced by contact splitting. However, it is not suitable to explain the phenomena of high friction in insects. Considering the contribution of viscosity, the estimated friction based on the hydrodynamic lubrication model cannot still explain the phenomenon that insects attach well on the vertical surfaces without sliding. Then the experimental measurement directly revealed that non-Newtonian shear-thinning property of the secretions leads to static friction (top in Figure 3C) (Dirks et al., 2010; Drechsler and Federle 2006). Besides, the boundary lubrication theory was also proposed, which can enhance the friction through increasing direct contacts between the adhesive organs and target substrates with decreasing the thickness of the fluid layer (bottom in Figure 3C) (Spikes 1996, 2000). Although different principles of wet adhesion were proposed for specific adhesion cases, they can only interpret the corresponding adhesion behaviors to a certain extent.

Furthermore, these principles still need confirmation and improvement through experimental studies. For instance, the basic components and the corresponding physicochemical properties (e.g., viscosity and surface tension) and wettability of different contact surfaces should be explored in detail. Then, the morphology of adhesive elements on adhesive pads, effective thickness of secretion layers between adhesive pads and contact surfaces, dependence of viscosity to shear rate, as well as dynamic molecular ordering at the contact surfaces should be compared. Based on the above-mentioned measurements, it is feasible to give a more accurate explanation.

Knowledge regarding the function of adhesive liquids, involved in the typical biological wet adhesive surfaces, is focused on discussing the effect of viscosity, composition, and wettability of substrates by adhesive liquids. For instance, it was revealed that adhesive liquid on the adhesive pads of flies, ants, and locusts is consisting of two separate liquid phases (i.e., hydrophobic and hydrophilic phases) (Federle et al., 2002). The thickness of the adhesive liquid layer and its viscosity were also estimated (Federle et al., 2002). Federle et al. (2010) suggested that the two-phasic nature of the adhesive liquid increases the ability to withstand shear forces because of the fine-tuning of rheological properties (Dirks et al., 2010). Gorb et al. (2014) estimated the viscosity of adhesive liquids in a beetle (lipid-based fluid) and a fly (water-lipid-based microemulsion) using a microrheological technique, which suggested that the contact formation time of spatula-like terminal adhesive elements is proportional to the viscosity (Peisker et al., 2014). In addition, Lämmerhofer et al. (2016) reported that adhesive liquids showed species-dependent complex hydrocarbon mixing ratios, which may function together with proteins and carbohydrates to mediate the physicochemical properties and the corresponding wet adhesion (Gerhardt et al., 2016). Most recently, a fluid secretion layer with a thickness of 10-20 nm between the adhesive pad of ladybird beetle and the substrate was observed using cryo-scanning electron microscopy (Hosoda et al., 2021). The generated adhesion was proportional to the square root of the dispersive part of the surface free energy on the contact substrate, which proves that intermolecular forces mainly contribute to the adhesion. However, the university of this principle needs further confirmation through choosing more adhesive systems. To understand the chemical principle under wet adhesion, a surface-sensitive analytical technique (e.g., SFG spectroscopy) was employed to investigate the interacts between the fluid secretion layer and three substrates with different wettabilities (Fowler et al., 2021a). The results show a positive relationship between the hydrocarbon ordering ratio at the contact interface and substrate hydrophobicity (Figure 3D), which also correlates with the enhanced adhesion through increasing hydrophobicity of the substrates. Thus, the dynamic molecular ordering at the contact surface also plays a significant role in mediating wet adhesion. To date, various principles were proposed to explain wet adhesion phenomena. Considering the complexity of wet adhesion including the components of secretions and the specific environmental surface, we need to use several principles or to develop new concepts to understand wet adhesion.

### Artificial wet adhesive surfaces

**Figure 4:**
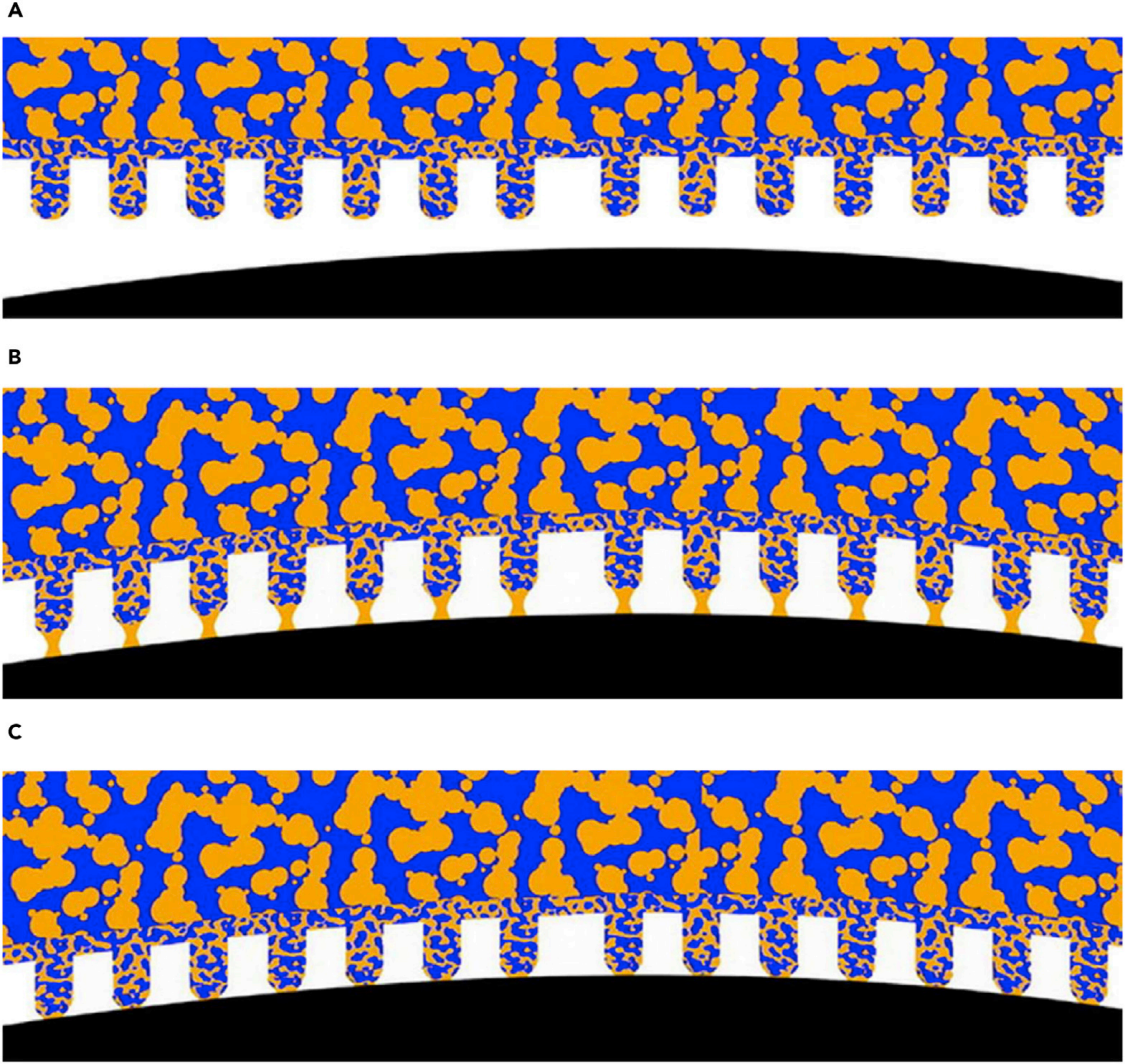
Artificial wet adhesive surfaces combined with adhesive elements (i.e., porous nanofibers) and a thin layer of adhesive liquid (i.e., mineral oil) (A) Porous nanofibers on adhesive pads are approached to the contact surface. (B) Formation of liquid bridges between the nanofibers and the contact surface. (C) Formation of solid-solid contact mediated by liquid bridges. The contact surface and adhesive liquid are represented by the black and orange colors, respectively.

Taking advantage of the mediation of attachment and detachment, insects can adapt their adhesive pads to achieve flexible locomotion, suitable fixation, and accurate predation. Inspired by this phenomenon, reversible wet adhesive surfaces under various external stimuli have been constructed in recent years. For example, the reversible underwater adhesion was achieved owing to the switching between hydrophobic-hydrophilic of N-isopropylacrylamide under the stimuli of temperature (Zhao et al., 2017). Combining hygroscopicity with *in situ* photocuring, fast and strong underwater adhesion was also achieved (Zhou et al., 2021). It is suggested that reversible wet adhesion can be obtained through the rational regulation of adhesive surfaces and adhesive liquids. For artificial wet adhesive surfaces, Xue et al. (2015) developed fibrillar pads consisting of continuous pores through which liquid (in this work mineral oil) can be delivered to the contact surfaces for adhesion management (Figure 4) (Xue et al., 2015). The results suggested that the synergistic interaction of capillarity and humidity-induced pad softening lead to the increase of the adhesion force. However, up to now, only a few research about artificial liquids with components and physicochemical properties similar to biological adhesive liquids and their effect on wet adhesion were reported. Owing to the complexity and variety of biological adhesive liquids (Federle et al., 2002; Gerhardt et al., 2016), it is rather difficult to determine the role of each of these components. In practice, we usually focus on investigating the role of one or several components. A biomimetic emulsion with three kinds of components such as squalane, deuterated stearic acid, and D_2_O was prepared to explore the contribution to bulk and surface-specific properties in mediating wet adhesion (Fowler et al., 2021b). First, similar hydrocarbon ordering on the substrates with different wettabilities as fluid secretions in beetles was observed through SFG spectra. Then, the shear-thinning profile was also obtained through rheological testing, which indicates the ability to increase the traction forces during locomotion. Thus, it is meaningful to identify artificial adhesive liquids with similar components and properties as biological adhesive liquids by adjusting the mixing rations of lipid and water to investigate the corresponding physicochemical properties (e.g., viscosity and surface tension) and wettability on different contact surfaces. Moreover, the wet adhesion properties (e.g., adhesive force and adhesion energy) of the model adhesive surfaces (e.g., adhesive surfaces with spongy nanopores and porous nanofibers) combined with artificial adhesive liquids (e.g., a range of simple model liquids and complex liquids) should be explored systematically in the future.

(A–C) Reproduced with permission (Xue et al., 2015). Copyright 2015, Macmillan Publishers Limited

### Supply of liquids to contact surfaces of adhesive systems

First, the adhesive element is defined as the minimum adhesion structure (e.g., nanofibers) in close contact with the substrate. During the locomotion of insects, secretions must be supplied to the tips of the adhesive elements to keep wet adhesion in a normal operation state. Thus, it is necessary to explore the delivery of adhesive liquids through nanochannels to the surface (Andersson et al., 2017; Haridasan and Sathian 2021; Nazari et al., 2020; Wang et al., 2018b; Zhang et al., 2019a). Up to now, a few papers have reported the transport of secretions in insects. For instance, Bauchhenss in 1979 suggested that the adhesive liquid of the fly is released through channels under the pressure exerted by its weight (Bauchhenss 1979). Later, Gorb (1998) observed channels with 0.1–0.4 μm diameter in the seta shaft on the adhesive pads of flies (Gorb 1998). The secretion rate of adhesive liquids in adhesive pads of cockroaches and stick insects was quantified by Dirks et al. (2011), showing that the secretion of adhesive liquids is controlled by negative capillary pressures (Dirks and Federle 2011b). Although the transporting principles of secretions in insects were not clear enough, there is no doubt that it is a feasible way to mediate wet adhesion by adjusting the adhesive liquids through the nanochannels. Recently, the concept of quantum-confined superfluid has been proposed for rapid mass transport in confined nanochannels, which is enthalpy-driven ordered fluid and shows no energy loss (Chen et al., 2021c; Wen et al., 2018; Zhang and Jiang 2019; Zhang et al., 2019b). This concept can explain the ultrafast neural signal transmission in living organisms, such as the transformation of various biological information in smell and vision and so on. The above-mentioned mass mainly includes water and various ions. So, it is meaningful to investigate whether the transport of adhesive liquids in the biological nanochannels has similar effects. If so, the supply of secretions will benefit from the transport in nanochannels. Moreover, the supply of artificial adhesive liquids through nanochannels and the effect on wet adhesion need to be explored.

### Future directions

Later in discussion, we discuss the concept of bio-inspired wet adhesion control with liquids. First, our knowledge regarding the actual function of adhesive liquids in mediating adhesion is still very limited, which requires us to further explore their underlying working principles. Second, bio-inspired artificial adhesive liquids should be developed and employed for wet adhesion combined with model adhesive surfaces. Third, the supply of artificial adhesive liquids through nanochannels still lacks research and leaves large room for investigating the effect on wet adhesion.

**Figure 5:**
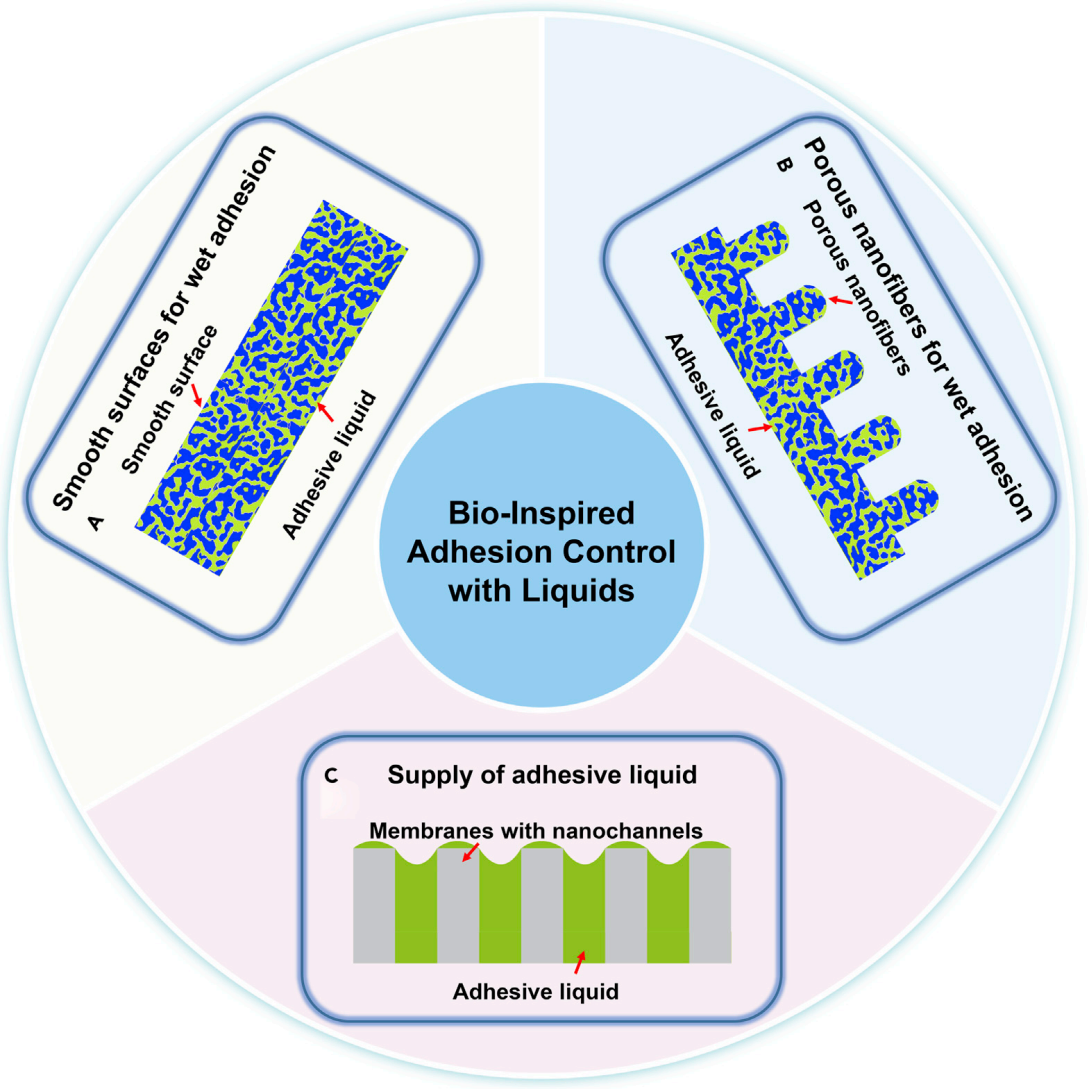
Schematic illustration of artificial adhesive surfaces using liquids (A) Smooth adhesive surfaces with spongy nanopores combined with artificial adhesive liquids. (B) Porous nanofibers combined with artificial adhesive liquids. (C) Membranes with nanochannels for supplying adhesive liquids. The adhesive surfaces with and without nanofibers, membrane with nanochannels and adhesive liquids are represented by the blue, gray and green colors, respectively.

To meet the aforementioned challenges related to the adhesion control with liquids, feasible routes for further research are provided using the model adhesive surfaces and liquids (Figure 5). For example, we can design model adhesive surfaces with various geometric parameters for investigating adhesion performance firstly. As we focus on the effects of the adhesive liquids on wet adhesion, representative adhesive surfaces should be developed. In nature, the adhesive elements on adhesive pads are usually nanofibers with different tip shapes, such as flat, convex, discoidal, concave, spatular nanofibers. To make sure a sufficient supply of liquids during the repeated attachment and detachment, the adhesive surfaces are required to be porous. Thus, a limited number of robust and easily accessible model adhesive surfaces, such as a smooth adhesive surface with spongy nanopores and an adhesive surface with porous nanofibers can be selected to keep the parameter space to be covered manageable (Figures 5A and 5B).

Considering the complex adhesive secretions, we can select one component which shows similar adhesion properties at the beginning. A screening of wet adhesion using the prepared model adhesive surfaces can be carried out with a range of simple model liquids that contain only one component. The model liquids are potential components of more complex liquids that may mimic natural adhesive secretions. The impact of the simple model liquids on the mechanical properties and adhesion of the model adhesive surfaces can be explored. Subsequently, selected mixtures of the simple model liquids with systematically varied compositions can also be investigated for their role in wet adhesion (Xue et al., 2015).

Furthermore, for insects, timely supplementation of adhesive fluids is required to ensure sufficient wet adhesion on various substrates during locomotion (Bauchhenss 1979; Dirks and Federle 2011b; Gorb 1998). However, the research on this aspect is insufficient in both the biological and artificial systems, owing to the complexity of adhesive fluids. Thus, it is necessary to investigate the transport of adhesive liquids in nanochannels for supply. As shown in Figure 5C, the supply of the adhesive liquids through transporting in the artificial nanochannels, as well as the effect on wet adhesion, should be investigated. Meanwhile, the underlying mechanisms of wet adhesion with liquids can be further explored using finite element analysis or fluid dynamics simulations. Based on the research, artificial adhesive surfaces with specific functionalities are anticipated to be developed by inducing the liquids, which will promote applications in different fields. Notably, with the development of wet adhesive surfaces and the corresponding characterization techniques, more rational routes can also be developed for further research.

## Conclusion

Insects generate adhesion on various substrates using the secreted liquids playing an important role in adhesion control. The in-depth understanding of the wet adhesion control using liquids can not only reveal the underlying functional mechanisms but also provide guidance for the rational construction of bio-inspired wet adhesive surfaces. Herein, we review the concept of bio-inspired wet adhesion control using liquids. We provided information about biological and artificial wet adhesive surfaces and discuss possibilities to transport adhesive liquids through nanochannels for the timely supply to the contact surfaces. In the future, the main tasks of the ongoing research and development will be 1) to achieve rational wet adhesion control by liquids and 2) to further construct artificial wet adhesive surfaces with specific functionalities. First of all, the rational regulation of wet adhesion of model surfaces with the selected model liquids needs to be explored, and the corresponding theoretical models need to be established. Secondly, the supply of artificial adhesive liquids through nanochannels and their effect on wet adhesion should be studied. The herewith proposed direction in surface science will lead to the development of artificial wet adhesive surfaces with specific functionalities and long-term operation times.
